# Cases

**Published:** 1847-12

**Authors:** T. C. Osborne

**Affiliations:** Erie, Ala.


Art. V.—*Cases.* By Dr. T. C. Osborne, of Erie, Ala.

*Death from Over-eating.—Autopsy.* In September, 1846, a stout-bodied negro, twenty years of age, previously unhealthy, ceased to labor on account of an œdematous affection of the inferior extremities, preceded by pains and immobility of the knee joints. In the following February, having exposed himself carelessly on several occasions to inclement weather, anasarca became general, and ascites, acute cephalalgia, bulimia, and pain in the right hypochondrium, were in a short time superadded. Mercurials, drastic purgatives, and iodine were administered, and by the 20th of March he was able to resume his place in the field; the morbid appetite, however, continued to occasion so much distress that he declared an intention to appease it if it should cost him his life. Accordingly, on the night of the 10th of May, he stole and ate the week's allowance of a healthy negro man. Next morning he went out to work as usual, but getting thirsty, stopped at a stagnant puddle and drank water until the stomach became painfully distended, which was followed in a short time by convulsions and apoplectic coma. When I arrived, four hours afterwards, he was in the following condition: extremities cold and trembling; pulse 70, natural; respiration prolonged and deep without stertor; immobility of the eyes and contracted pupils; heedless of noise or violence. After 40 ounces of blood had been taken, by venesection and cups to the temple, he awoke, complained of pain in the head and right side, and, grasping the penis, hastily directed a full stream of clear urine against the wall. I mention this circumstance because, during the remainder of his sickness, although able to walk about and converse rationally, the dejections of urine and fæces passed involuntarily.

May 27th. Emaciation—delirium—convulsions—death.

*Autopsy*, six hours after death. When the cranium was opened a large quantity of thin, dark, offensive blood escaped. On the convex surface of the left anterior lobe of the brain, the arachnoid membrane exhibited a circumscribed patch, two inches in diameter, of diffuse redness. Cerebrum strikingly blanched. Right lateral ventricle contained a small quantity of bloody serum.

*Thorax.* Organs normal.

*Abdomen.* Liver pale and indurated. On the convexity of the right lobe there was a white circular cicatrix as large as a quarter of a dollar, and of cartilaginous consistence. Two others were found on the left lobe. Near the cicatrix on the right lobe the liver and diaphragm were united by adhesion, requiring for their separation very delicate dissection. Gall bladder filled with black bile.

*Stomach.* Two kinds of discoloration pervaded the mucous membrane of this organ—one a large patch at the great extremity, of a deep livid or purple color; the other consisted of several small patches of intense redness, distributed in an arborescent and speckled manner over the pyloric extremity. The mucous texture at the great curvature was softened and covered by a thick coat of tenacious mucous. Other abdominal viscera healthy.

*Purpura Hemorrhagica.* The history of any fatal case of disease, unaccompanied by the post-mortem revelations, is comparatively valueless; but in as much as the design in the present instance is to illustrate the efficacy of an agent in the cure of a malady, by adducing subsequent examples of its successful application, the subjoined case, it is believed, will be found instructive.

*Case 1st.* During a tedious convalescence from a protracted tertian fever, Miss ———, aged 12 years, in easy circumstances and well grown, was observed, on the 5th of August, 1842, to have an eruption of stigmata on the extremities, unattended with fever. These continued to multiply rapidly and coalesce, changing their color from a deep red to a dark purple, up to the 7th, the time of my first visit. The surface was now spotted everywhere with petechiæ and vibices, irregular in figure and size, leaving pale and flaccid intervals, which rendered the appearance of the patient singularly frightful and forbidding. Blotched face, ink-stained appearance of the lips, buccal membrane, fauces, and nasal cavities; dark, spongy, blood-oozing gums; furred tongue; accelerated pulse; slight fever, and headache. The appetite and bowels regular and good. Patient amusing herself out of doors when I arrived.

*Treatment.* Ordered her immediately to bed; a tepid pediluvium; a purge of calomel and jalap, to be followed in a few hours by castor oil and spts. turpentine.

8th. Bowels freely opened by medicines, and dejections of a bilious appearance; nausea, vomiting, fever, thirst, epigastric tenderness and pain, huried respiration, quick pulse, intense cephalalgia, hemorrhage from the stomach, mouth and nose.

An orifice was made in a vein of the arm, but from the rapid sinking of the pulse was quickly closed again. The surface around the incision became in a few moments ecchymosed. Cold water to the head; blister on the region of the stomach; barks, acid, wine, cinnamon water, apple-ade, turpentine enemata, and mustard foot bath.

9th. The blistered surface was elevated and filled with fluid blood; increased hemorrhage; wild and anxious expression; constant tossing; great thirst; ardent fever; incessant efforts at vomiting, the matter ejected consisting of water and occasionally black blood. Every thing taken by the mouth and anus rejected instantly.

10th. Ecchymosed eyes—delirium—death.

*Case 2d.* A male infant five months old, the offspring of indigent and unhealthy parents, had a severe attack of pneumonia on the 1st of October, 1843, but on the 9th was thought to be convalescent. Ten days afterward it was taken with purpura hemorrhagica of an asthenic and aggravated character.

A pail full of cold water was immediately dashed on the naked infant, and ordered to be repeated night and morning; a decoction of bark acidulated with elixir vitriol three times daily; port wine. In illustration of the magical manner in which this course arrested the frightful progress of the disease, it is sufficient to state, that on the 22d, three days after it was commenced, the patient was considered well.

The insertion of the following letter is due to my friend, Dr. Norman, of Wilson county Tennessee, to whom I am indebted for a knowledge of the powers of the cold dash in this most perplexing disease. The letter was intended to reach me during my attendance on the case first detailed, and is dated 8th August, 1842:

Mrs. ——— informs me you have a case of purpura. I have had two cases and treated both successfully. It is a rare disease in our section of country, and I know you will not think me too officious in giving you my treatment. This consisted in the very free use of *muriatic acid*, the *cold dash*, and mild aperients. One case occurred as the sequel to an attack of fever; the patient had been slowly convalescing for three weeks, when, rather suddenly, he was taken with hemorrhage from the gums, and petechiæ from head to foot. Being treated as above stated, he was in a week quite well The only circumstance worth your notice is the *freedom* with which the acid may be used, and the *efficacy* of the cold dash. I have taken this liberty because I was at a loss myself, and would gladly have received any information on the subject.

Very truly, your friend, THOS. NORMAN.

Dr. A. Puckett, late of Beardstown, Tennessee, in a letter dated about the same time, states that a man of athletic frame was attacked with purpura hemorrhagica shortly after recovering from intermittent fever. In addition to the symptoms of congestion and phlogosis in this case, there was a very considerable œdema of the extremities—"the fingers and toes stood in a strut." But under the liberal use of the treatment recommended by Dr. Norman, his convalescence was rapid and complete.

From these cases and much reading on the subject—having attentively examined the history of every case that I can find recorded—I have arrived at the following conclusions respecting this obscure affection:

1st. That purpura hemorrhagica is sequent upon some other disease or morbid condition of the system.

2d. That the altered condition of the blood is the proximate cause of the disease, which is itself dependent upon a morbid state of the ganglionic system.

3d. That there are evidently two varieties of the disease; the sthenic, or congestive; and the asthenic, or adynamic.

4th. That it bears no relation whatever to scorbutus, except in that condition of the system observed in typhus, yellow fever, pestis, and cholera, as well as in this disease which facilitates the appearances known as the characteristics of purpura hemorrhagica.

6th. That the phlogistic symptoms observed in purpura are the result of a combat between the capillaries of a healthy organ, and the chemically changed blood.

7th. That the cold dash is applicable in every case.

*Worms found in the Heart and Bloodvessels of a Dog; Symptoms of Hydrophobia.* Drs. Jennings, Brown, Thorp, and myself recently made a post-mortem examination of a dog that died of supposed hydrophobia. The symptoms were those characterizing that disease, and so well marked as to alarm and mislead the entire community. He was one of those much valued hounds, used in hunting negroes; an old dog, whose health had been good; fat, ordinarily remarkably peaceable; but after his attack, the disposition and appearance of the animal were completely changed. He snapped at every living thing near him, assailing dogs, or bitches, acquaintances or strangers indiscriminately; exhibited dread of water; emaciation; convulsions; and death ensued in ten or fifteen days from the time he was first observed to be diseased.

*Autopsy.*—The stomach and other abdominal organs were healthy; but the large blood-vessels were found filled with worms; the heart contained of these at least one hundred. The appearance of the worms was remarkably similar, differing only in length. They were round, white, tapering towards the tail, and from half an inch to four inches long, the thickness varying from one-fourth of a line to one line. I did not note as carefully as I ought the form of the head and mouth, and I believe the same is true of the gentlemen who assisted me in the examination.

Among the number of the bitten dogs was a pointer puppy which alone showed any symptoms of the same disease. It had two fits, and during the second leaped from a second story window, and was so injured that its owner had it killed. No dissection was made in this case.

Were the convulsions in these cases excited by worms? In the first case, I think there can be no question that they were, and it is to be regretted that a *sectio* was not made of the body of the second, to determine the fact whether worms existed in it also. Was the disease *hydrophobia?* I should say it was not, from the fact that of the number of animals bitten by the blood-hound but a single one exhibited signs of disease afterwards. Is it not probable that many of the cases regarded as hydrophobic, are in reality diseases of a different character, resulting from irritation in some part of the system? And may not worms occasionally be the cause of this irritation? The subject appears to me worthy of investigation, and repeated examinations of the bodies of these animals might lead to valuable conclusions.

October, 1847.

